# A pilot study evaluating the feasibility of assessing undergraduate pharmacy and medical students interprofessional collaboration during an online interprofessional education intervention about hospital discharge

**DOI:** 10.1186/s12909-023-04557-x

**Published:** 2023-08-21

**Authors:** Hailah Almoghirah, Jan Illing, Mahdi Nazar, Hamde Nazar

**Affiliations:** 1 Newcastle University, Newcastle-upon-Tyne, UK; 2 King Saud University, Riyadh, Saudi Arabia; 3 RCSI University of Medicine and Health Sciences, Dublin, Ireland; 4 Brampton Medical Practice, Carlisle, UK

**Keywords:** Interprofessional education, Undergraduate, Assessment, Communication skills, Behavioural change, Patient outcome

## Abstract

**Background:**

Interprofessional education (IPE) has been identified as a strategy towards improving competence at interprofessional working and collaboration within teams. Entrustable professional activities (EPAs) provide a framework for translating competencies into elements of clinical practice, some of which in healthcare are inherently interprofessional. However, it is challenging to reconcile that entrustment decisions about student competence in an interprofessional activity are made about an individual without considering the dynamics and tensions between interprofessional team members and the task itself. This can influence students’ development and demonstration of competence at interprofessional collaboration.

**Methods:**

In this study, undergraduate medical and pharmacy students worked in pairs online (Zoom) to undertake the hospital discharge process (a professional activity reliant on interprofessional collaboration) for a simulated patient, producing a hospital discharge letter and completing a consultation with the simulated patient. The online sessions were recorded and interprofessional behaviours were assessed using a validated scale completed by an interprofessional assessment team. Students undertook this IPE intervention three times after receiving feedback and a period of reflection each time.

**Results:**

Eighteen students participated across the entire intervention and 27 one-hour online IPE sessions were completed and recorded. Students demonstrated statistically significant improvements in interprofessional behaviours across the three iterations (p < 0.05 for all the sessions). The discharge letter students produced also improved over the three sessions (p = 0.01). Students found the educational sessions useful and relevant.

**Conclusion:**

This online IPE intervention provided the students with an authentic opportunity to work collaboratively. At the end of each iteration, students received feedback about their work as a team and about the discharge letter, helping students to reflect and purposefully develop their performance. The IPE intervention with this assessment strategy is feasible and allows student development to be captured but has proved to be time and resource intensive.

**Supplementary Information:**

The online version contains supplementary material available at 10.1186/s12909-023-04557-x.

## Background

Interprofessional education (IPE) has been identified as a strategy towards improving interprofessional working and collaboration, and thereby improves patient outcomes [1, 2].

IPE is a statutory requirement for many undergraduate training programmes, e.g., in the United Kingdom (UK), IPE is stipulated in the standards for education and training by the General Medical Council, General Pharmaceutical Council and Nursing and Midwifery Council [3–5].

However, conducting authentic and effective IPE is complex. Many challenges have been identified which include finding a convenient time and place that suits all trainee professionals involved, coordinating between different health professional curricula [6, 7], and the lack of different professional programmes within the same institution to take part in IPE [8].

Reeves and colleagues emphasise the importance of developing IPE experiences that are authentic, high in fidelity (corresponding to the degree of realism of simulation created with the use of equipment, setting and scenario) [9], include a patient and involve measuring outcomes [1, 8]. Such experiences should allow students to develop interprofessional competencies such as better communication, collaboration, and coordination of care. Assessing the impact of IPE and these interprofessional competencies are also challenging, as there is no specific IPE intervention and assessment fit for all purposes and for all professionals [8, 10]. Robust assessment approaches are those which best measure the change in student behaviour objectively after IPE rather than relying on self-assessment, which is limited by its subjective nature [8].

Entrustable Professional Activities (EPAs) offer the opportunity to translate competencies into health tasks or responsibilities that a trainee can perform with a level of supervision that correlates to the level of entrustment in that trainee’s competence [11]. EPAs are best suited to individuals rather than teams in health care which frequently change in composition. However, Ten Cate and Pool recently contest that much work conducted in healthcare relies on interprofessional collaboration, meaning many EPAs are by their very nature interprofessional [12].

Patient admission and discharge planning was identified as an EPA that can often require interprofessional collaboration. Hospital discharge relies on effective communication, coordination and collaboration between healthcare settings, thereby is inherently multiprofessional in nature [12–15]. One component in the process is the discharge letter or summary, which is a key document produced by hospital staff. It records information about admission, hospital stay, discharge and aftercare and needs to be transmitted to primary care providers to ensure continuity of care. In most cases, this record is completed by doctors or nurses. However, recent research has shown a benefit when there is input from a pharmacist. For example, completing a medicines reconciliation prior to discharge reduces the risk of any drug related errors prior to discharge [16]. Also, general practitioners reported that poor discharge letters and summaries can lead to extra work needed from them and a negative patient experience [17].

In this study, we describe an IPE intervention framed around the EPA of hospital discharge planning for medical and pharmacy undergraduate students. We feasibility test the delivery of this intervention online, which overcomes some of the challenges of synchronising time, space and accessibility of students [6]. Additionally, to select an appropriate measure of student competence at interprofessional collaboration, we will use our decision aid produced as part of a systematic review about the evidence for validated tools for assessing performance at IPE [10].

## Method

We conducted a prospective pilot study assessing undergraduate student interprofessional collaboration during an online IPE intervention. The primary goal was to test the feasibility of delivering this IPE intervention and using a validated tool to assess student performance. A feasibility study is one which is conducted on a small scale, aiming to test and check if a future large-scale study is worthwhile [18].

Mixed methods have been employed in this study and the Strengthening The Reporting of Observational studies in Epidemiology (STROBE) checklist has been used to frame the reporting [19]. (See appendix S1) All methods were carried out in accordance with relevant guidelines and regulations.

### Participant recruitment

In one institution based in England, students from the final year (5th year) of the Bachelor of Medicine and Bachelor of Surgery (MBBS) (n = 332) and final year (4th year) and 3rd year of the Master of Pharmacy (MPharm) degree (n = 152) were identified as suitable participants to be involved. Students at these levels of study had already engaged in some forms of IPE and had gained the fundamental knowledge and skills required to conduct a hospital discharge for a patient. Students were recruited via an email invitation including a participant information sheet that was sent by the respective programme leads. A reminder email invitation was sent after two weeks. Participation was voluntary but was incentivised with a £50 voucher. Informed consent was obtained from all subjects prior to commencing the study. The recommended sample size for a feasibility study is 20 to 25 participants and this is what we aimed for in this study [20].

### IPE intervention design

The Guideline for Reporting Evidence-Based Educational Interventions and Teaching (GREET) checklist was used to best report the intervention [21]. (See appendix S2) The IPE intervention was informed by two EPAs from the medical and pharmaceutical literature [22, 23]. (See appendix S3) These were identified from Haines S et al. (2017) *and* Obeso V et al. (2017) and are relevant to support safe and effective hospital discharge [22, 23].

The undergraduate medical and pharmacy students were tasked to undertake the following tasks online in a one-hour session:

- Review patient hospital notes to identify the patient needs as they are discharged back home;
- create an appropriate discharge letter to facilitate safe and effective handover to primary care.
- undertake a consultation with a simulated patient to discuss the care plan and manage the discharge.

Real patient scenarios were sourced, and anonymized, from a local secondary care hospital. A clinical pharmacist and teaching academic were tasked to identify patients who were due to be discharged. The patient hospital notes were reviewed and then used to form the cases for the IPE sessions. The cases were between 15 and 40 pages long. A real-life patient scenario was desired to ensure the simulation was as authentic as possible and the simulated patient was a paid actor.

The online session was recorded with consent. The recording and the created discharge letters were submitted to an assessment team comprised of an academic pharmacist and a practicing general practitioner (GP).

### IPE intervention pre-pilot

The intervention was pre-piloted with one medical student and one pharmacy student to test the online delivery, check timing and to ensure it was well received.

Students were provided with the patient case and tasked to undertake the activities in the one-hour online session. After the pilot session, the students reported positively about the session, stating there was enough time to complete the tasks in the time allocated. However, they suggested receiving the patient case earlier, as they needed most of the one hour to complete the discharge letter and consult with the patient. Both students found it was relevant and likely to help them prepare for practice.

### IPE intervention pilot

No changes were made to the content or structure of the IPE intervention because of the pre-pilot test.

The medical and pharmacy students were randomly assigned in pairs to work together over three iterations. The three sessions were scheduled at least two-weeks apart to allow for the student work to be assessed and feedback provided. Students were provided with a brief (2-min) recorded presentation that described the aim, learning outcomes and tasks of the session.

In response to the feedback from the pilot, the students were provided with the patient scenario one-day before their scheduled session. The patient scenarios were provided in order from least to most complex (over the three iterations) according to the number of comorbidities and patient needs. All material provided to the students for the sessions are outlined in Table 1 and further described in the GREET checklist.

**Table 1:** Material provided for students and simulated patient

Material for students				Material for simulated patient
*Pilot session*	***1st Set of Sessions***	***2nd Set of sessions***	***3rd Set of sessions***	
At the time of the pilot session, both students received an email including: A pre-recorded introduction video, which contained instructions to be viewed before they started the IPE intervention. Consent forms to be completed and returned before the session started. The anonymized patient case, with biochemical laboratory results. Discharge letter template to be completed and returned after the session directly. (See appendix S4) The Zoom meeting link.	A reminder was sent to participants a week before the session.			Simulated patient was provided with the Zoom meeting links and a brief about the patient case. This consisted of important information for the simulated patient such as: Instructions The patient’s medical conditions The patient’s medication history A list of any allergies Reasons for admission Two suggested questions to ask the students.
	One day before the sessions, students received an email including:			
	Introduction video to view before the session. Consent forms to be completed and returned before their first session. Anonymized patient case with biochemical laboratory results. Discharge letter template to be completed and returned after the session directly. The Zoom meeting link.	Anonymized patient case with biochemical laboratory results. Discharge letter template to be completed and returned after the session directly. The Zoom meeting link.	Anonymized patient case with biochemical laboratory results. Discharge letter template to be completed and returned after the session directly. The Zoom meeting link.	

### IPE assessment

A multi-modality approach to assessment was taken to gain a better understanding of student learning. The assessment strategies were selected based on their capacity to assess across the levels of the Kirkpatrick/Barr model [24]. (See appendix S5) The Interprofessional Professionalism Assessment (IPA) tool was used to assess behavioural change, the discharge letter (proxy measure) was used to assess benefit to patient and student self-assessment was used to assess the learner’s reaction.

#### IPA tool

From the decision aid in our previous work [10], we identified that the Interprofessional Professionalism Assessment tool (IPA) [25] has good reliability and validity in assessing performance at the individual level. It measures the behaviours of Interprofessional professionalism and communication skills across 6 domains: Communication; Respect; Altruism and Caring; Excellence; Ethics and Accountability (See appendix S6). The assessment team were tasked to watch together a one-hour recording of students’ activity and use the IPA tool to assess their performance. The assessment team had to reach consensus on their scoring and agree on the IPA qualitative comments that were provided as feedback to students.

#### Discharge letters

The student produced discharge letters were also marked by the assessment team using a rubric that assessed across three domains: completeness, quality, and presentation (See appendix S7). Each domain was rated out of five, creating a score from 0 to 15. A model discharge letter was used as a guide (the one produced in the hospital for the real patient) and consensus on student scores had to be reached by the assessment team.

#### Student self-assessment

The IPA scores, qualitative feedback and marked discharge letters were shared with the students via email following each IPE session. Students were asked to reflect on their performance and the feedback that they had received. They were prompted to answer and note down their responses to the following three questions and return this back to the researcher. This feedback strategy was adopted to help students focus on areas for improvement as recommended by Boud et al. [26].

1. What did you do well?
2. What areas did you find challenging?
3. What areas do you plan to improve on?

### Data analysis

Data from the IPA tool and discharge letters were first entered into a Microsoft Excel version 2108 spreadsheet then exported to IBM SPSS Statistic (Statistical Package for Social Sciences) (Version 27) [27] to be analysed.

Data was analysed for students who completed all three sessions. This meant data for one student who only completed one session was excluded.

#### IPA tool

We analysed change in the IPA score from intervention one to intervention two and from intervention two to intervention three using the Mixed ANOVA. Then, we conducted an IPA subgroup analysis using the Mixed ANOVA for the following domains: Communication; Respect; Altruism and Caring; Excellence and Accountability [25].

#### Discharge letters

We analysed the improvement in scores for the discharge letters across completeness, quality, and presentation from intervention one to intervention two and from intervention two to intervention three using the Mixed ANOVA.

#### Student self-assessment

Student answers to the three questions were analysed using content analysis and verbatim quotes identified to illustrate key themes [28].

### Ethical approval

Ethical approved was obtained from the University ethics committee (reference number: 5299/2020) and the study was reviewed at the medical school Research Management Group where a permission to proceed was gained.

## Results

This IPE intervention was carried out from February 2021 until May 2021. In total, 23 students agreed to take part: 12 pharmacy students and 11 medical students. Two medical students later withdrew before the study started. One pharmacy student also withdrew before it started, and another one after the first session. One could not continue as the sessions were full and no medical students were available to work with him. After withdrawals, 18 students continued the study to completion: nine pharmacy students and nine medical students. Most of participants in both professions were female (n = 14, 77.7%). There were three medical and one pharmacy student who were male. Each student completed three sessions either with the same student or different student depending on the mutual availability of the students. We conducted a total of 27, one-hour online IPE sessions (9 one-hour recordings per IPE iteration (n = 3)). The assessment team completed 54 IPA tools (one for each student pair across the 27 IPE sessions) and evaluated 27 discharge letters.

### IPA scores

**Fig. 1:**
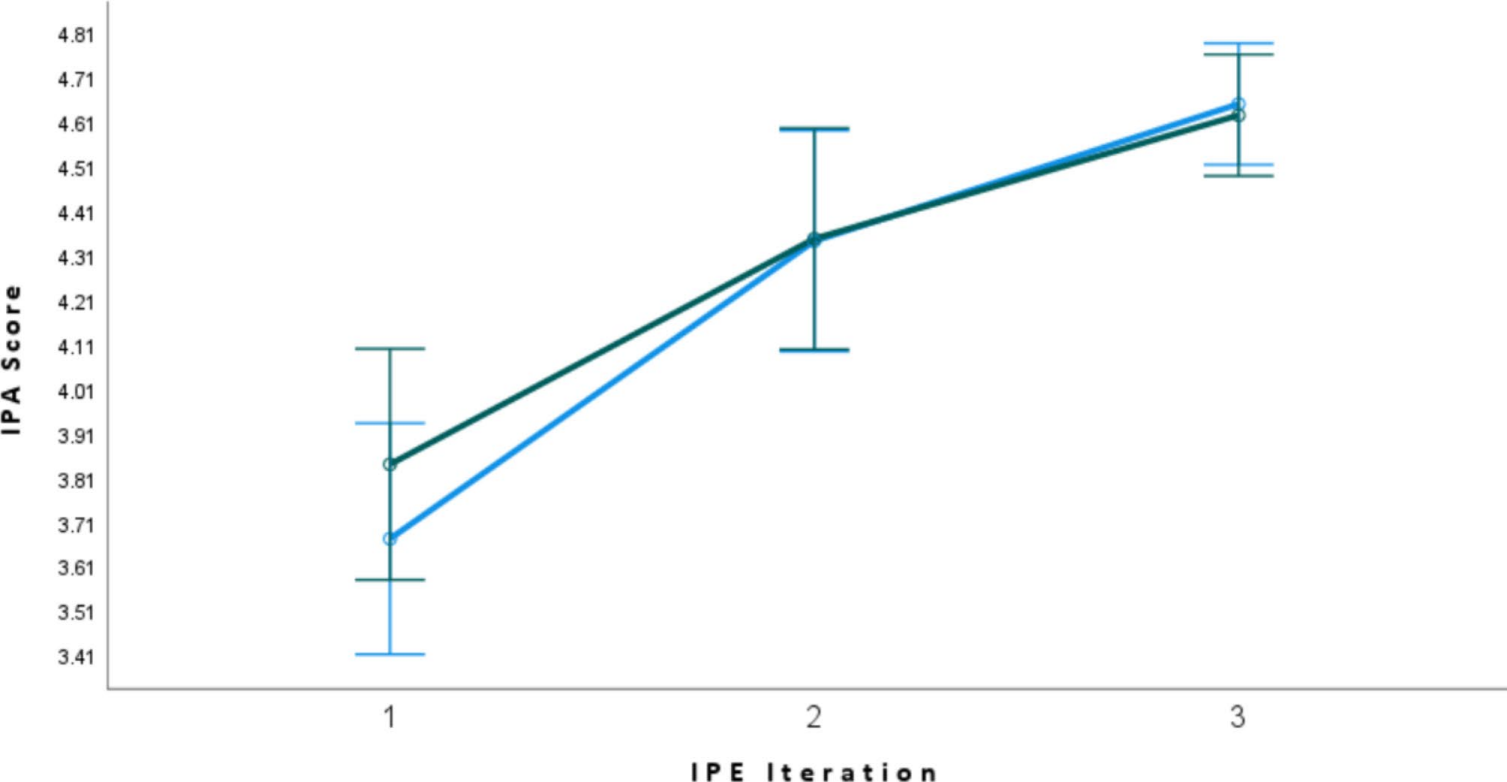
The IPA scores (estimated marginal mean; error bars 95%Cl) for pharmacy (green) and medical (blue) students across the three sessions

The IPA scores improved with statistical significance from session one to session two and from session two to session three. (See Fig. 1; Table 2) Student performance significantly improved across all five domains of the IPA over the three IPE iterations. (p < 0.05 for all the sessions). There was no statistically significant difference in improved performance between the medical and pharmacy students (p = 0.578). (See appendix S8)

**Table 2:** The comparison of IPA scores between the three IPE sessions using mixed ANOVA analysis

IPA scores compared between IPE sessions	Mean Difference	Std. Error	Sig.^a^	95% Confidence Interval for Difference	
				Lower Bound	Upper Bound
1 vs. 2	− 0.588^*^	0.120	0.000	− 0.909	− 0.267
1 vs. 3	− 0.881^*^	0.109	0.000	-1.173	− 0.590
2 vs1	0.588^*^	0.120	0.000	0.267	0.909
2 vs. 3	− 0.293^*^	0.079	0.006	− 0.504	− 0.081
3 vs. 1	0.881^*^	0.109	0.000	0.590	1.173
3 vs. 2	0.293^*^	0.079	0.006	0.081	0.504

Based on estimated marginal means

*. The mean difference is significant at the 0.05 level

a. Adjustment for multiple comparisons: Bonferroni

### Discharge letter scores

**Fig. 2:**
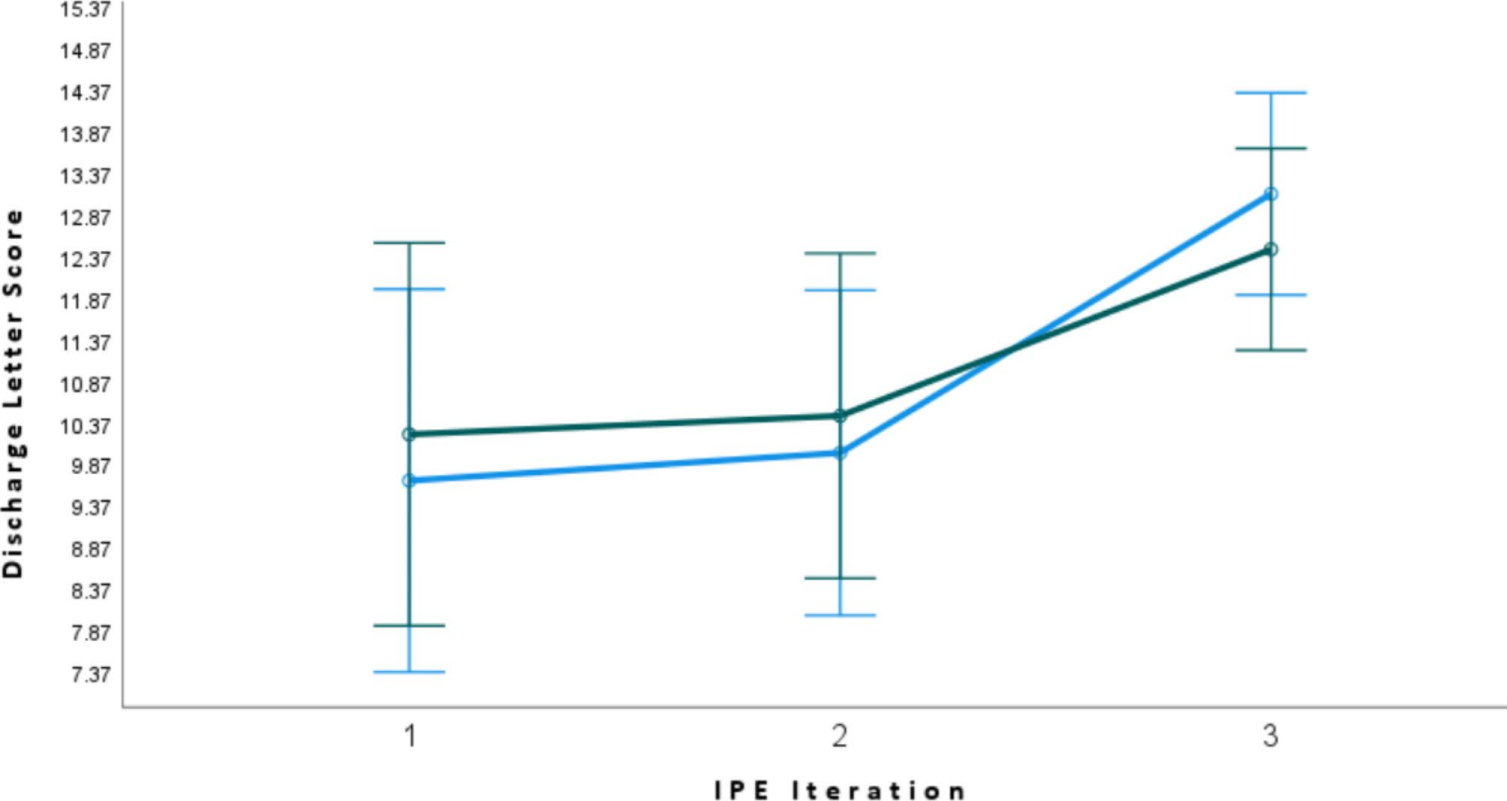
The scores of the discharge letters (estimated marginal mean; error bars 95%Cl) for pharmacy (green) and medical (blue) students across the three sessions

The scores of the discharge letters were statistically significantly improved over the three IPE iterations (p = 0.01) There was no statistically significant difference in improved discharge letters between the medical and pharmacy students (p = 0.681) (See Fig. 2 and appendix S9).

### Student self-assessment

The analysis of student reflective feedback identified the following themes: communication with the other student, the patient, and the task. In Table 3 are illustrative quotes.

**Table 3:** Content analysis for feedback questions

What did you do well	What areas did you find challenging?	What areas do you plan to improve on?
**Communication with student**	**Communication with student**	Communication with student
*“Worked together well”* (medical student, year 5, intervention 1). *" Got better team collaboration and working together and understanding the roles of each other”* (medical student, year 5, intervention 3).	*“We had not delegated clearly what each person was going to talk about“(*pharmacy student, year 4, intervention 1) *" I didn’t know what to expect of the pharmacy student”* (medical student, year 5, intervention 3).	*“Ask the pharmacy student about their background and what points are important to them“*(medical student, year 5, intervention 1) *“Continue to involve the med student in my thought processes and decisions”* (pharmacy student, year 3, intervention 2)
**Communication with patient**	**Communication with patient**	Communication with patient
*" Ensuring that they understood what was important, and what the next steps were. Including safety-net advice“(*medical student, year 5, intervention 1) *“Explaining their medication to the patient and asking them how they felt about their medication”* (pharmacy students, year 3, intervention 1)	*“Nervous when they ask me a question I have not prepared fully for”* (pharmacy students, year 3, intervention 2) *“Responding to their queries in a way that they can understand "* (medical student, year 5, intervention 3)	*“Be more of a conversation with the patient than a lecture”* (pharmacy students, year 3, intervention 1) *“Focus the consultation with the patient better with the structure”* (medical student, year 5, intervention 2)
**The task**	**The task**	The Task
*“Ensured appropriate care“(*medical student, year 5, intervention 1). *“More confident relaying a treatment plan”* (pharmacy students, year 3, intervention 2)	*“Difficult to read the entire case fully“(*medical student, year 5, intervention 2) *“Summarizing and trying not to miss anything that might be important”* (pharmacy students, year 3, intervention 3)	*“End up with decently written discharge letters. “(*medical student, year 5, intervention 3) *“I think it was really important to repeat the sessions to try and get a hold of it*“(pharmacy student, year 4, intervention 3)

Collaboration with another healthcare student was identified as challenging but improved as students gained experience across the IPE iterations. Students reflected not knowing each other’s roles or areas of expertise being problematic.

Students reported that they thought that their communication with the patient was generally patient-centred but found it challenging when they had to answer patient questions about their discharge.

The task of discharge was reported to be unfamiliar, and challenges included lack of familiarity with patient notes, clinical management and specific knowledge about certain medications and potential interactions.

## Discussion

We have found this IPE intervention focused on hospital discharge to be feasible with undergraduate pharmacy and medical students, and the assessment approach captured student improvement in IPE behaviours using the IPA tool. The third iteration showed a statistically significant improvement. The discharge letter, being used as a proxy for a patient outcome, also improved over the IPE iterations. Students found the educational sessions useful and relevant.

It is positive to see that student performance measured with the IPA improved across the three sessions. The performance at the discharge letter also sharply improved particularly from the second to third sessions, indicating the importance of cognitive load [29]. The long patient notes to read and digest, the preparation of a discharge letter and then a consultation with a patient may have presented the students’ working memory with significant intrinsic and/or extrinsic information [29]. The improvement in discharge letter in the third session may indicate that the students had learnt how to best manage the load and focus their efforts. Before the second and third iteration, students were asked to reflect on the assessors’ feedback from the IPA tool they received and identify areas for improvement. This technique of closing the loop, meant students were actively considering their learning and development. This metacognition was also likely to help students focus on areas for improvement [27, 31].

The strength of this intervention was that it provided students from different professions an opportunity to work collaboratively on an authentic clinical process with a simulated patient. The online format meant the planning and scheduling was not limited by synchronizing timetables, room availability and capacity and presence of facilitators. The recording of the sessions meant assessment, especially by two assessors, was more flexible to arrange and manage. Also, the recent accelerated progress over the past two years with online educational delivery, has meant many challenges, e.g., problems with connectivity, reported in earlier studies have been reduced [30]. This intervention and evaluation drew upon the Kirkpatrick/Barr evaluation model [24] with defined outcomes meaning students received feedback across the levels of the model.

Lastly, healthcare delivery, transformed during the COVID-19 pandemic, is expected to continue to make use of digital platforms and technology going forward. Our IPE intervention, making use of digital platforms for professional and patient communication and consultation, is likely to mimic and prepare undergraduate students and trainees for contemporary practice in the post-COVID era.

Other research has identified that simulation based interprofessional interventions were found to have a positive effect in students’ perceptions and understanding of each other responsibilities [30, 32−33]. However, the outcomes reported for these experiences were limited to measuring students’ perceptions, attitude or knowledge not their behaviours. Other literature about online IPE interventions has also involved measuring student perception, attitude or knowledge mainly through the use of self-assessment tools [30, 34−37]. However, these studies articulated an ambition in future research to measure student behavioural improvement and the impact on patient outcome [35] Our study has achieved these ambitions.

Most significantly, our work adds to the debate about the relationship between EPAs and IPE [12]. We have shown that EPAs can be used to frame an educational intervention around a professional activity that requires interprofessional collaboration. Students must acknowledge, understand and navigate the division of labour and the functionality mediating artifacts such as the patient notes and discharge letter. Our assessment approach provides the opportunity to capture individual student performance within a team that is changing (mimicking teams in healthcare). This longitudinal and textured perspective of a student’s interprofessional competence will be helpful to make entrustment decisions for this professional activity. If a student has been able to identify (through reflecting on individual feedback), how they may better manage the dynamics within a team environment towards achieving a collective goal, then they are in a better position to demonstrate competence and secure entrustment to undertake that interprofessional activity with decreasing levels of supervision [12].

Although there were many strengths of this intervention, it is important to acknowledge that it was resource and time intensive. The sessions were one-hour long and the assessors spent around two hours assessing each student pair using the IPA tool, providing feedback, and assessing the discharge letter. Going forward, it might be possible to train students to use the assessment tool such that students conduct self- and peer-assessments, for a portfolio in a formative capacity, and a final summative session could be where academic and clinical staff time is invested towards informing entrustment decisions. Another limitation is that the patient cases varied in complexity. Ideally, the cases should have been similar. In this study, the cases became more complex with each iteration. Nonetheless, improvements in performance and learning were captured. There is a potential that if we had used cases at a similar level of complexity, we may have found an even greater improvement in learning.

A third limitation is that students were recruited via an email invitation that was sent by the respective programme leads. Participants may have felt they needed to participate at the request of a programme lead. In future, other academics that are not linked to the programme, could send the information to the students. Another limitation of the study was the reliance on students volunteering even thought they were incentivized. Student volunteers have been shown to be more intrinsically motivated [38] and therefore the intervention should be tested on a diverse population of students. This research was also limited by the small number of participants, recruited from just two programmes from one institution. However, we have compensated by capturing, analysing, and triangulating data from different sources to best investigate our research question and produce the findings. Lastly, we did not collect data from the simulated patient about their perceptions of interprofessional and effective care delivery which could be an area for further research.

The general approach to designing and assessing an IPE intervention in this study offers an evidence-based and pedagogically informed roadmap for other educators to apply in any other IPE environments and /or with any other health professions. A clinical task or process that benefits from interprofessional working can be deconstructed using the concept of EPAs. This means that the designed intervention will be based on contemporary clinical practice and offers students an authentic learning experience. A pluralistic assessment approach and use of an evidence-based tool (where this exists) will mean students receive targeted feedback on their performance to help them learn and develop. The online platform is a powerful medium to be exploited where in-person educational interventions are often logistically challenged, especially now, when virtual and digital healthcare delivery is likely to become more commonplace.

## Conclusions

We designed and implemented an online IPE intervention that simulated real-life practice. We were also able to demonstrate that student interprofessional collaboration improved significantly over three iterations of this intervention. Students reported the intervention to be useful and relevant to their future practice. Future studies are required to determine the scalability of this intervention given the recognised resource implications. Further work could explore the potential to train students in the assessment approach to allay some of these concerns and investigate the viability of using such individual performance measures to inform entrustment decisions about activities that are inherently interprofessional in nature.

### Electronic supplementary material

Below is the link to the electronic supplementary material.

Supplementary Material 1

## Data Availability

The datasets used and/or analysed during the current study are available from the corresponding author on reasonable request.

## References

[1] Reeves S, Boet S, Zierler B, Kitto S (2015). Interprofessional Education and Practice Guide No. 3: evaluating interprofessional education. J Interprof Care.

[2] Hammick M, Freeth D, Koppel I, Reeves S, Barr H (2007). A best evidence systematic review of interprofessional education: BEME Guide no. 9. Med Teach.

[3] General Pharmaceutical Council (2019). Consultation on initial education and training Standards for pharmacists.

[4] General Medical Council. Domain 3: communication partnership and teamwork. 2019. https://www.gmc-uk.org/ethic al-guida nce/ethic al-guida nce-for-docto rs/good-medic al-pract ice/domai n-3—communicat ion-partn ershi p-and-teamwork. Accessed November 2, 2019.

[5] Nursing and Midwifery Council (2008). Standards to support Learning and Assessment in Practice: NMC Standards for mentors, practice Teachers and teacher.

[6] Interprofessional Education Collaborative Expert Panel (2011). Core competencies for interprofessional collaborative practice: report of an expert panel.

[7] O’Keefe M, Henderson A, Chick R (2017). Defining a set of common interprofessional learning competencies for health profession students. Med Teach.

[8] Reeves S, Fletcher S, Barr H (2016). A BEME systematic review of the effects of interprofessional education: BEME Guide No. 39. Med Teach.

[9] Carey J, Rossler K (2023). The how when why of High Fidelity Simulation. StatPearls.

[10] Almoghirah H, Nazar H, Illing J (2021). Assessment tools in pre-licensure interprofessional education: a systematic review, quality appraisal and narrative synthesis. Med Educ.

[11] Ten Cate O (2013). Nuts and bolts of Entrustable Professional Activities. J Grad Med Educ.

[12] Ten Cate O, Pool IA (2020). The viability of interprofessional entrustable professional activities. Adv Health Sci Educ Theory Pract.

[13] Angley M, Ponniah A, Spurling L (2011). Feasibility and timeliness of Alternatives to Post-Discharge Home Medicines Reviews for High‐Risk Patients. J Pharm Pract Res.

[14] Hesselink G, Schoonhoven L, Barach P (2012). Improving patient handovers from hospital to primary care. Ann Intern Med.

[15] Medication Safety in Transitions of Care (2019). Licence: CC BY-NC-SA 3.0 IGO.

[16] Phatak A, Prusi R, Ward B (2015). Impact of pharmacist involvement in the transitional care of high-risk patients through medication reconciliation, medication education, and postdischarge call-backs (IPITCH Study). J Hosp Med.

[17] Weetman K, Dale J, Spencer R, Scott E, Schnurr S (2020). GP perspectives on hospital discharge letters: an interview and focus group study. BJGP Open.

[18] Eldridge S, Lancaster G, Campbell M (2016). Defining feasibility and Pilot Studies in Preparation for Randomised controlled trials: development of a conceptual Framework. PLoS ONE.

[19] Von Elm E, Altman DG, Egger M (2008). The strengthening the reporting of Observational Studies in Epidemiology (STROBE) statement: guidelines for reporting observational studies. J Clin Epidemiol.

[20] Lancaster G. Pilot and feasibility studies come of age! Pilot Feasibility Stud. 2015;1(1). 10.1186/2055-5784-1-1 10.1186/2055-5784-1-1 PMC5842886 29611687

[21] Phillips A, Lewis L, McEvoy M, et al. Development and validation of the guideline for reporting evidence-based practice educational interventions and teaching (GREET). BMC Med Educ. 2016;16(1). 10.1186/s12909-016-0759-1 10.1186/s12909-016-0759-1 PMC5011880 27599967

[22] Haines S, Pittenger A, Stolte S (2017). Core Entrustable Professional Activities for New Pharmacy Graduates. Am J Pharm Educ.

[23] Obeso V, Brown D, Aiyer M (2017). Core EPAs for entering Residency Pilot Program. Toolkits for the 13 Core Entrustable Professional Activities for entering Residency.

[24] Freeth D, Hammick M, Koppel I, Reeves S, Barr H (2002). A critical review of evaluations of Interprofessional Education.

[25] Frost J, Hammer D, Nunez L (2019). The intersection of professionalism and interprofessional care: development and initial testing of the interprofessional professionalism assessment (IPA). J Interprof Care.

[26] Boud D, Molloy E (2013). Rethinking models of feedback for learning: the challenge of design. Assessment & Evaluation in Higher Education.

[27] IBM Corp. Released 2020. IBM SPSS Statistics for Windows, Version 27.0. Armonk, NY: IBM Corp.

[28] Kolbe RH, Burnett MS (1991). Content-analysis research: an examination of applications with directives for improving research reliability and objectivity. J Consumer R.

[29] Young J, Van Merrienboer J, Durning S, Ten Cate O (2014). Cognitive load theory: implications for medical education: AMEE Guide No. 86. Med Teach.

[30] O’Shea M, Reeves N, Bialocerkowski A, Cardell E. Using simulation-based learning to provide interprofessional education in diabetes to nutrition and dietetics and exercise physiology students through telehealth. Adv Simul. 2019;4(S1). 10.1186/s41077-019-0116-7 10.1186/s41077-019-0116-7 PMC6923831 31890319

[31] Rogers G, Thistlethwaite J, Anderson E (2016). International consensus statement on the assessment of interprofessional learning outcomes. Med Teach.

[32] Thompson B, Bratzler D, Fisher M, Torres A, Faculty E, Sparks R (2016). Working together: using a unique approach to evaluate an interactive and clinic-based longitudinal interprofessional education experience with 13 professions. J Interprof Care.

[33] Alinier G, Harwood C, Harwood P (2014). Immersive clinical Simulation in Undergraduate Health Care Interprofessional Education: knowledge and perceptions. Clin Simul Nurs.

[34] Robertson B, McDermott C, Star J, Lewin L, Spell N (2021). Synchronous virtual interprofessional education focused on discharge planning. J Interprof Educ Pract.

[35] Djukic M, Adams J, Fulmer T (2015). E-Learning with virtual teammates: a novel approach to interprofessional education. J Interprof Care.

[36] Abdelaziz A, Mansour T, Alkhadragy R, Abdel Nasser A, Hasnain M (2021). Challenges to Interprofessional Education: will e-Learning be the magical Stick?. Adv Med Educ Pract.

[37] Claiborne D, Durgampudi P, Patel P, Akpinar-Elci M (2021). Dental hygiene and public health students’ perception of an online interprofessional education applied learning activity. J Dent Educ.

[38] Gegenfurtner A, Könings K, Kosmajac N, Gebhardt M (2016). Voluntary or mandatory training participation as a moderator in the relationship between goal orientations and transfer of training. Int J Train Dev.

