# Supplementary material for: A pilot study evaluating the feasibility of assessing undergraduate pharmacy and medical students interprofessional collaboration during an online interprofessional education intervention about hospital discharge
Source: BMC Med Educ. 2023 Aug 21;23:589. doi: 10.1186/s12909-023-04557-x (PMC10441699; doi:10.1186/s12909-023-04557-x)
Supplement: Supplementary file 1 — Supplementary Material 1 [file 12909_2023_4557_MOESM1_ESM.docx]

**Supplementary information**

Table of contents

Appendix S1: The Strengthening the reporting of observational studies in epidemiology (STROBE) checklist …………………………………………………………………………………………………………………….….………………2-3

Appendix S2: The Guideline for Reporting Evidence-Based Educational Interventions and Teaching (GREET) checklist …………………………………………………………..………………………………………….…….……………4-8

Appendix S3: Table to show the Entrustable Professional Activities relevant to hospital discharge…………………………………………………………………………………………………………………………………..….....9

Appendix S4: Discharge Letter Template…..…………………………………………………………………..….……….10-12

Appendix S5: The Kirkpatrick/Barr model and assessment strategy employed.………………………..……..13

Appendix S6: The IPA tool adopted from Frost *et al.* ………………………………………………….………………15-20

Appendix S7: Discharge letter’s rubric used by assessment team ……………………………………..….……21-22

Appendix S8: The Difference of IPA scores between professions.…………………….……………..…………..….14

Appendix S9: Discharge letter scores improvement over the three IPE sessions and between professions using mixed ANOVA analysis. ……...……………………………..……………………….…………….…….....15

**Appendix S1:** The Strengthening the reporting of observational studies in epidemiology (STROBE) checklist

Checklist of items that should be included in reports of cohort studies

|  | Item No | Recommendation | Page No |
| --- | --- | --- | --- |
| **Title and abstract** | 1 | (*a*) Indicate the study’s design with a commonly used term in the title or the abstract | 1-2 |
|  |  | (*b*) Provide in the abstract an informative and balanced summary of what was done and what was found | 2 |
| Introduction | | | |
| Background/rationale | 2 | Explain the scientific background and rationale for the investigation being reported | 3 |
| Objectives | 3 | State specific objectives, including any prespecified hypotheses | 4 |
| Methods | | | |
| Study design | 4 | Present key elements of study design early in the paper | 4 |
| Setting | 5 | Describe the setting, locations, and relevant dates, including periods of recruitment, exposure, follow-up, and data collection | 4 |
| Participants | 6 | (*a*) Give the eligibility criteria, and the sources and methods of selection of participants. Describe methods of follow-up | 4 |
|  |  | (*b*) For matched studies, give matching criteria and number of exposed and unexposed | NA |
| Variables | 7 | Clearly define all outcomes, exposures, predictors, potential confounders, and effect modifiers. Give diagnostic criteria, if applicable | 6,7 |
| Data sources/ measurement | 8* | For each variable of interest, give sources of data and details of methods of assessment (measurement). Describe comparability of assessment methods if there is more than one group | 6,7 |
| Bias | 9 | Describe any efforts to address potential sources of bias | 5 |
| Study size | 10 | Explain how the study size was arrived at | 4 |
| Quantitative variables | 11 | Explain how quantitative variables were handled in the analyses. If applicable, describe which groupings were chosen and why | 6,7 |
| Statistical methods | 12 | (*a*) Describe all statistical methods, including those used to control for confounding | 7 |
|  |  | (*b*) Describe any methods used to examine subgroups and interactions | 7 |
|  |  | (*c*) Explain how missing data were addressed | 6 |
|  |  | (*d*) If applicable, explain how loss to follow-up was addressed | NA |
|  |  | (*e*) Describe any sensitivity analyses | NA |
| Results | | |  |
| Participants | 13* | (a) Report numbers of individuals at each stage of study—eg numbers potentially eligible, examined for eligibility, confirmed eligible, included in the study, completing follow-up, and analysed | 7 |
|  |  | (b) Give reasons for non-participation at each stage | 7 |
|  |  | (c) Consider use of a flow diagram | NA |
| Descriptive data | 14* | (a) Give characteristics of study participants (eg demographic, clinical, social) and information on exposures and potential confounders | 7 |
|  |  | (b) Indicate number of participants with missing data for each variable of interest | 7 |
|  |  | (c) Summarise follow-up time (eg, average and total amount) | NA |
| Outcome data | 15* | Report numbers of outcome events or summary measures over time | 7-8 |

| Main results | 16 | (*a*) Give unadjusted estimates and, if applicable, confounder-adjusted estimates and their precision (eg, 95% confidence interval). Make clear which confounders were adjusted for and why they were included | 7-8 |
| --- | --- | --- | --- |
|  |  | (*b*) Report category boundaries when continuous variables were categorized | NA |
|  |  | (*c*) If relevant, consider translating estimates of relative risk into absolute risk for a meaningful time period | NA |
| Other analyses | 17 | Report other analyses done—eg analyses of subgroups and interactions, and sensitivity analyses | 7 |
| Discussion | | | |
| Key results | 18 | Summarise key results with reference to study objectives | 9 |
| Limitations | 19 | Discuss limitations of the study, taking into account sources of potential bias or imprecision. Discuss both direction and magnitude of any potential bias | 10 |
| Interpretation | 20 | Give a cautious overall interpretation of results considering objectives, limitations, multiplicity of analyses, results from similar studies, and other relevant evidence | 9,10 |
| Generalisability | 21 | Discuss the generalisability (external validity) of the study results | NA |
| Other information | | | |
| Funding | 22 | Give the source of funding and the role of the funders for the present study and, if applicable, for the original study on which the present article is based | Funding information |

*Give information separately for exposed and unexposed groups.

**Note:** An Explanation and Elaboration article discusses each checklist item and gives methodological background and published examples of transparent reporting. The STROBE checklist is best used in conjunction with this article (freely available on the Web sites of PLoS Medicine at http://www.plosmedicine.org/, Annals of Internal Medicine at http://www.annals.org/, and Epidemiology at http://www.epidem.com/). Information on the STROBE Initiative is available at http://www.strobe-statement.org.

NA: Not applicable

**Appendix S2:** The Guideline for Reporting Evidence-Based Educational Interventions and Teaching (GREET) checklist

| **GREET 2015 checklist*** based upon the TIDieR guidance |  |  |
| --- | --- | --- |
| BRIEF NAME | | |
| 1. INTERVENTION:   We conducted a feasibility, prospective, cohort study. Undergraduate pharmacy and medical students were expected to undertake the following tasks online in a one-hour session:   - Review the patient case together, which were presented in the form of anonymized real hospital notes from a local secondary care hospital. - Collaboratively create a hospital discharge letter to facilitate safe and effective handover to primary care. - Have an online consultation with the simulated patient to discuss the care plan and manage the discharge.   The online session was recorded with consent, and the created discharge letters were submitted to an assessment team consisting of an academic pharmacist and a practicing general practitioner (GP). It is a mixed-methods prospective study. | |  |
| WHY - this educational process | |  |
| 1. THEORY: In the intervention, we used an Entrustable Professional Activities (EPAs) to develop a robust authentic IPE intervention and we employed an evidence-based approach for the assessment. Cognitive learning theory and closing the Loop were used to explain students' learning improvement. | |  |
| 1. LEARNING OBJECTIVES:   The learning objectives for the undergraduate medical and pharmacy students were to:   - Demonstrate interprofessional working whilst undertaking an online hospital discharge process; - Create an appropriate discharge letter for the patient transition, and - Undertake a consultation with a simulated patient to discuss and manage the transition home from hospital. | |  |
| 1. EBP CONTENT:   This authentic online interprofessional education was based on the major steps in the evidence-based practice procedure. First, we asked an answerable question about developing an online IPE intervention for undergraduate medical and pharmacy students focused on the hospital discharge process drawing on the relevant EPAs and developing an appropriate assessment strategy to measure IPE performance using a validated tool. Second, we searched for the best evidence in this field. Third, we appraised the finding to develop the IPE intervention and we used our previous publication to decide the assessment approach. Fourth, we applied this intervention for undergraduate medical and pharmacy students during three alterations. Fifth, we assessed this intervention using the assessment approach selected to identifying how to optimize the IPE intervention and assessment strategy to facilitate and capture student learning and development. | |  |
| WHAT | |  |
| 1. MATERIALS   Material provided before and during the study are described below. | |  |
| \| Material for students \| \| \| \| Material for simulated patient \| \| --- \| --- \| --- \| --- \| --- \| \| *Pilot session* \| ***1^st^ Set of***  ***Sessions*** \| ***2^nd^ Set of sessions*** \| ***3^rd^ Set of sessions*** \| \| At the time of the pilot session, both students received an email including:   - A pre-recorded introduction video, which contained instructions to be viewed before they started the IPE intervention. - Consent forms to be completed and returned before the session started. - The anonymized patient case, with biochemical laboratory results. - Discharge letter template to be completed and returned after the session directly. (See appendix S4) - The Zoom meeting link. \| A reminder was sent to participants a week before the session. \| \| \| Simulated patient was provided with the Zoom meeting links and a brief about the patient case.  This consisted of important information for the simulated patient such as:   - Instructions - The patient’s medical conditions - The patient’s medication history - A list of any allergies - Reasons for admission - Two suggested questions to ask the students. \| \| One day before the sessions, students received an email including: \| \| \| \| - Introduction video to view before the session. - Consent forms to be completed and returned before their first session - Anonymized patient case with biochemical laboratory results. - Discharge letter template to be completed and returned after the session directly. - The Zoom meeting link. \| - Anonymized patient case with biochemical laboratory results - Discharge letter template to be completed and returned after the session directly. - The Zoom meeting link. \| - Anonymized patient case with biochemical laboratory results - Discharge letter template to be completed and returned after the session directly. - The Zoom meeting link. \|  - The introduction video can be accessed through this link: <https://campus.recap.ncl.ac.uk/Panopto/Pages/Viewer.aspx?id=0a3d6850-0525-480a-8592-aceb01418244> - The patient cases, the biochemical laboratory and the simulation case scenarios are available upon request from the research team.  1. EDUCATIONAL STRATEGIES:   We conducted a total of 28, one-hour online IPE sessions using Zoom platform for undergraduate medical and pharmacy students. Each student completed three sessions. On each occasion the medical and pharmacy students were randomly assigned in pairs to work together. The patient cases were provided in order from least to most complex (over the three iterations) according to the number of comorbidities and patient needs. | |  |
| 7. INCENTIVES: Each student and the General Practitioner who helped in students' assessment were thanked for their time by receiving an Amazon voucher. 50£ Amazon voucher for each student and 500£ Amazon voucher for the General Practitioner. | |  |
| WHO PROVIDED | |  |
| 1. INSTRUCTORS:   In this educational intervention 4 instructors were involved.   \| **Instructor** \| **Professional discipline** \| **Teaching experience/expertise** \| **Role and Specific training provided to be able to teach the intervention.** \| \| --- \| --- \| --- \| --- \| \| Prof. Jan Illing \| Professor of Health Professions Education. \| >20 Years \| JI was involving on:   - Designing the project and supervising all the project stages. - Analysing the data. \| \| Dr. Hamde Nazar \| Senior Lecturer in pharmacy practice and a Senior Pharmacist. \| >10 Years \| HN was involving on designing the project and supervising all the project stages. In addition to:   - Help in identifying the patient cases from the hospital. - Help in Role-player booking process. - Evaluating 54 students using Interprofessional professionalism assessment (IPA) tool. - Evaluating 27 discharge letters. - Analysing the data. \| \| Dr. Mahdi Nazar \| General practitioner  Bachelor of Medicine and Member of the Royal College of General Practitioners. \| <10 years \| MN was involving in:   - Evaluating 54 students using IPA tool. - Evaluating 27 discharge letters. \| \| Hailah Almoghirah \| PhD researcher and demonstrator in clinical pharmacy. \| <10 years \| HA was involving in:   - Designing the project. - Sending the participation's invitation. - Contacting with the participants. - Preparing the materials - Arranging the sessions. - Supervising the sessions. - Providing the help when needing during the session - Conducting 9 focus groups with the participants. - Collect and analysing the data. \| | |  |
| HOW | |  |
| 1. DELIVERY:   The educational intervention was delivered online. During each session, students worked interprofessional as a team of two, one of each profession to conduct an online hospital discharge process, create an appropriate discharge letter for the patient transition and undertake a consultation with a simulated patient to discuss and manage the transition home from hospital. HA Supervised the sessions so the learners to instructors ration is 2:1 | |  |
| WHERE | |  |
| 1. ENVIRONMENT:   Each session was conducted using Zoom. Students used their own devices to join the sessions. | |  |
| WHEN and HOW MUCH | |  |
| 1. SCHEDULE:   This project was carried out from February 2021 until May 2021. Dates and times for the sessions were arranged with the students who agreed to participate using either email or Doodle Poll. The IPE intervention was undertaken three times. On each occasion the medical and pharmacy students were randomly assigned in pairs to work together. The three sessions were scheduled with at least two-weeks apart to allow for the student work to be assessed and feedback provided. Each session lasted for one-hour. | |  |
| 1. Describe the amount of time learners spent in face to face contact with instructors and any designated time spent in self-directed learning activities.   Students were provided with a brief (2-min) recorded presentation that described the aim, learning outcomes and tasks of the session. No reading materials were provided to the students before starting the sessions because students from both professions previously covered the discharge process and interprofessional education. | |  |
| PLANNED CHANGES | |  |
| 1. Did the educational intervention require specific adaptation for the learners?   The cases were for a real patients discharge previously from the hospital. So, for the purpose of the project, the dates in the patient cases were changed based on the date of the sessions to make it more reliable for the students. Also, a simulated case scenario was created from the original case to make it easier for the simulated patient to understand the case and play the role of the patient. | |  |
| UNPLANNED CHANGES | |  |
| 1. Was the educational intervention modified during the course of the study?   The intervention was piloted with one medical student and one pharmacy student to test the online delivery, check timing and to ensure it was well received. After the pilot session, an online meeting was conducted with both students to gain their feedback about the intervention, its acceptability, relevance, and time requirements. The students reported positively about the session, stating there was enough time to complete the tasks in the one-hour allocated. However, they suggested receiving the patient case earlier, as they needed most of the one hour to complete the discharge letter and consult with the patient. Both students found it was relevant and likely to help them prepare for practice. No changes were made to the content or structure of the IPE intervention as a consequence of the pilot. In response to the feedback from the pilot, the students were provided with the patient scenario one-day before their scheduled session. | |  |
| HOW WELL | |  |
| 1. ATTENDANCE: Describe the learner attendance, including how this was assessed and by whom. Describe any strategies that were used to facilitate attendance.   Student participation was noted through attending the sessions during the time they allocate to. HA attended each session to make sure that the students attended the session. | |  |
| 1. Describe any processes used to determine whether the materials (item 5) and the educational strategies (item 6) used in the educational intervention were delivered as originally planned.   All the sessions were recorded with permission. HA supervised all the sessions to make sure it is run as originally planned and that the students completed the tasks and provided a help when needed. | |  |
| 1. Describe the extent to which the number of sessions, their frequency, timing and duration for the educational intervention was delivered as scheduled (item 11).   The sessions were successfully completed as originally planned and no changes in frequency or duration were happen. For the timing, we needed in some sessions to reschedule the session's time due to unavailability of a participant. But in general, we managed to do that, and all the sessions were conducted successfully. | |  |

***based on the TIDieR guidance. We strongly recommend reading this statement in conjunction with the GREET 2015 explanation and elaboration paper for important clarifications on all the items. If relevant, we also recommend reading the TIDieR guidance (Hoffman et al. 2014)**

**Appendix S3:** The Entrustable Professional Activities relevant to hospital discharge

AACP: American Association of Colleges of Pharmacy, AAMC: Association of American Medical Colleges.

| Specific to pharmacists (AACP) | | Specific to medical doctors (AAMC) | |
| --- | --- | --- | --- |
| Entrustable Professional Activity | **Example supporting tasks** | **Entrustable Professional Activity** | **Example supporting tasks** |
| Implement a care plan in collaboration with the patient, caregivers, and other health professionals (Patient Care Provider Domain). | - Write a note that documents the findings, recommendations, and plan from a patient encounter. - Educate a patient regarding the appropriate use of a new medication, device to administer a medication, or self-monitoring test. | **Give or receive a patient handover to transition care responsibility.** | - Document and update an electronic handover tool and apply this to deliver a structured verbal handover. - Conduct handover using communication strategies known to minimize threats to transition of care. - Demonstrate respect for patient’s privacy and confidentiality. |
| Collaborate as a member of an interprofessional team (Interprofessional Team Member Domain). | - Contribute medication-related expertise to the team’s work. - Use setting appropriate communication skills when interacting with others. - Use consensus building strategies to develop a shared plan of action. | **Collaborate as a member of an interprofessional team.** | - Identify team members’ roles and responsibilities and seek help from other members of the team to optimize health care delivery. - Include team members, listen attentively, and adjust communication content and style to align with team-member needs. - Establish and maintain a climate of mutual respect, dignity, integrity, and trust. |

- Haines S, Pittenger A, Stolte S et al. Core Entrustable Professional Activities for New Pharmacy Graduates. *Am J Pharm Educ*. 2017;81(1):S2. doi:10.5688/ajpe811s2
- Obeso V, Brown D, Aiyer M et al. eds.; Core EPAs for Entering Residency Pilot Program. Toolkits for the 13 Core Entrustable Professional Activities for Entering Residency. Washington, DC: *Association of American Medical Colleges*; 2017

**Appendix S4:** Discharge Letter Template

**NHS**

The Newcastle Upon Tyne Hospitals

NHS Foundation Trust

.........your name………

……….address………………………………………………………………………………………………………....

Royal Victoria Infirmary

Queen Victoria Road

Newcastle upon Tyne

NE1 4LP

<Tel:01912825031>

[www.newcastle-hospitals.org.uk](http://www.newcastle-hospitals.org.uk)

visit date : ………………..

Typed on : ………………..

Lead Clinican : Dr. XXX

Pt. Name : ……………………………….. DOB: …………………..

NHS: …………………………………. MRN: …………………...

…………………………………………………………………………………………………………………………………………………………………………………………..

**Diagnosis:**

**………………………………………**

**……………………………………….**

**……………………………………….**

**Problem List:**

……………………………………………..

……………………………………………..

……………………………………………..

……………………………………………..

……………………………………………..

**Allergies:**

……………………………………………..

……………………………………………..

**Clinical details:**

……………………………………………………………………………………………………………………………………………………………………………………………………………………………………………………………………………………………………………………………………………………………………………………………………………………………………………………………………………………………………………………………………………………………………………………………………………………………………………………………………………………………………………………………………………………………………………………………………………………………………………………………………………………………………………………………………………………………………………………………………………………………………………………………………………………………………………………………………………

**Investigations performed:**

……………………………………………..

……………………………………………..

……………………………………………..

**Operation and procedures:**

……………………………………………..

……………………………………………..

……………………………………………..

**Discharge Medication:**

…………………………………………………………………………………………GP to continue? …....

…………………………………………………………………………………………GP to continue? …....

…………………………………………………………………………………………GP to continue? …....

…………………………………………………………………………………………GP to continue? …....

…………………………………………………………………………………………GP to continue? …....

…………………………………………………………………………………………GP to continue? …....

…………………………………………………………………………………………GP to continue? …....

…………………………………………………………………………………………GP to continue? …....

…………………………………………………………………………………………GP to continue? …....

…………………………………………………………………………………………GP to continue? …....

…………………………………………………………………………………………GP to continue? …....

…………………………………………………………………………………………GP to continue? …....

…………………………………………………………………………………………GP to continue? …....

…………………………………………………………………………………………GP to continue? …....

**Change to medication:**

…………………………………………………………………………………………….. …………………………………………………………………………………………….. …………………………………………………………………………………………….. …………………………………………………………………………………………….. …………………………………………………………………………………………….. ……………………………………………………………………………………………..

**Outpatient appointment:**

**Action for GP**

**………………………………………………………………………………………………………………………………………………………………………………………………………………………………………………………………………………………………………………………………………………………………………………………………………………………………………………………………………**

**Your sincerely**

**………………………………..**

**………………………………..**

**Appendix S5:** The Kirkpatrick/Barr model and assessment strategy employed

| Level | Outcomes | Assessment strategy |
| --- | --- | --- |
| 1 | Learner’s reaction | Student self-assessment |
| 2a. | Modification of attitudes/perceptions | Unreported in this study |
| 2b. | Acquisition of knowledge/skills | Unreported in this study |
| 3 | Behavioural change | IPA tool |
| 4a. | Change in organisational practice | Not assessed |
| 4b. | Benefits to patients, families, and communities | Discharge letter (proxy measure) |

**Appendix S6:** The IPA tool adopted from Frost *et al.*

*** Frost J, Hammer D, Nunez L, et al. The intersection of professionalism and interprofessional care: development and initial testing of the interprofessional professionalism assessment (IPA). *J Interprof Care*. 2019;33(1):102-115.


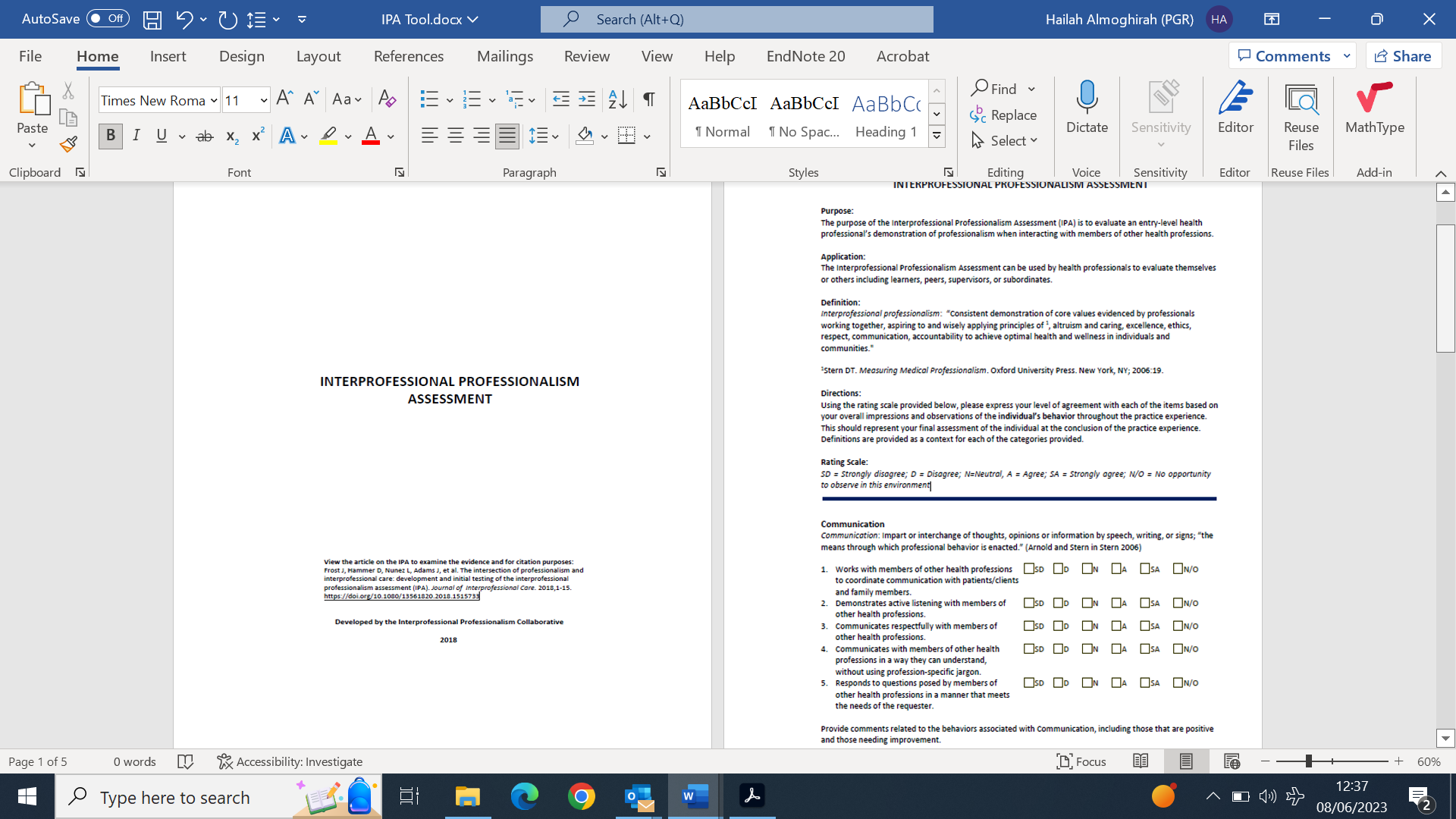


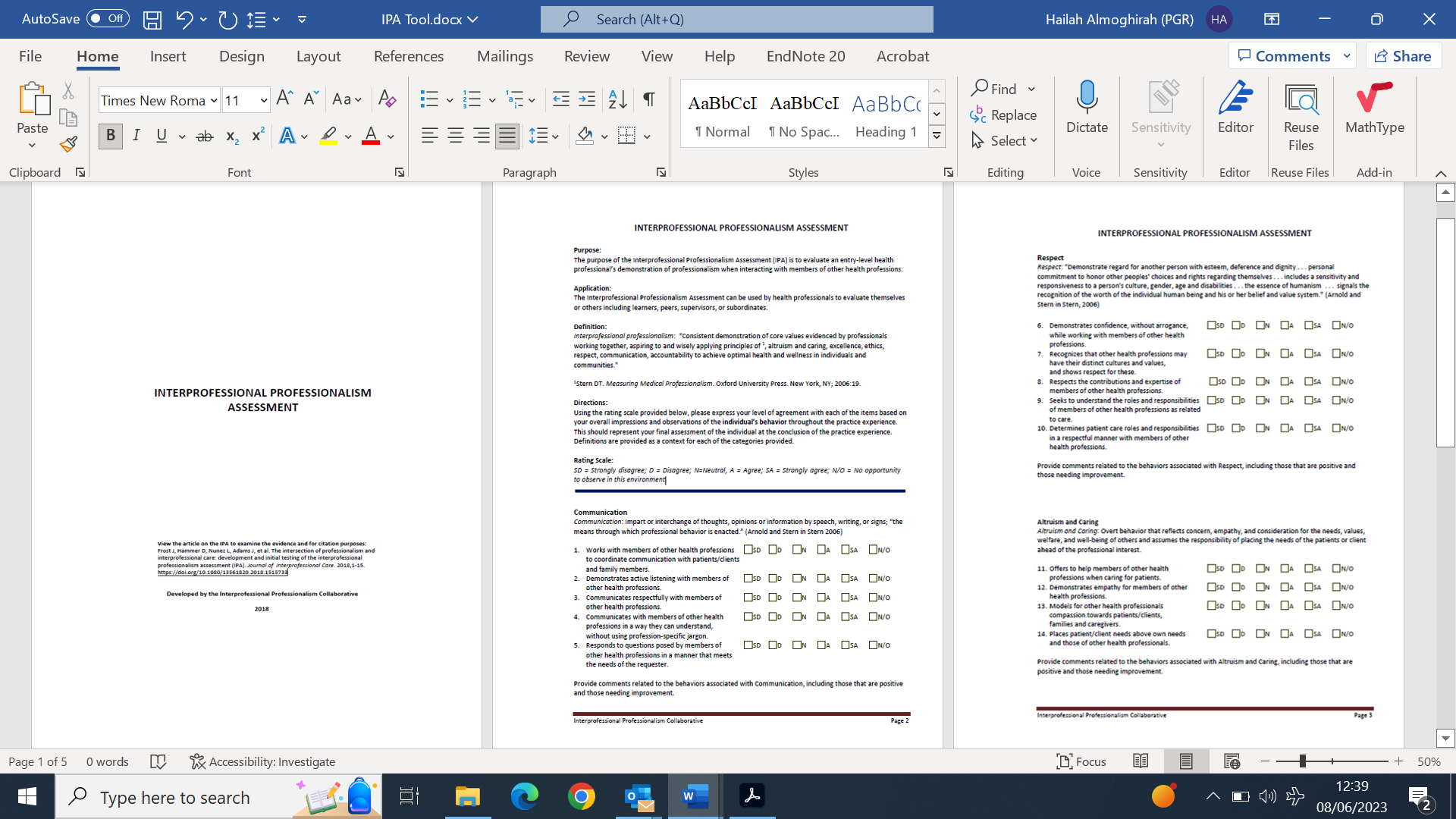


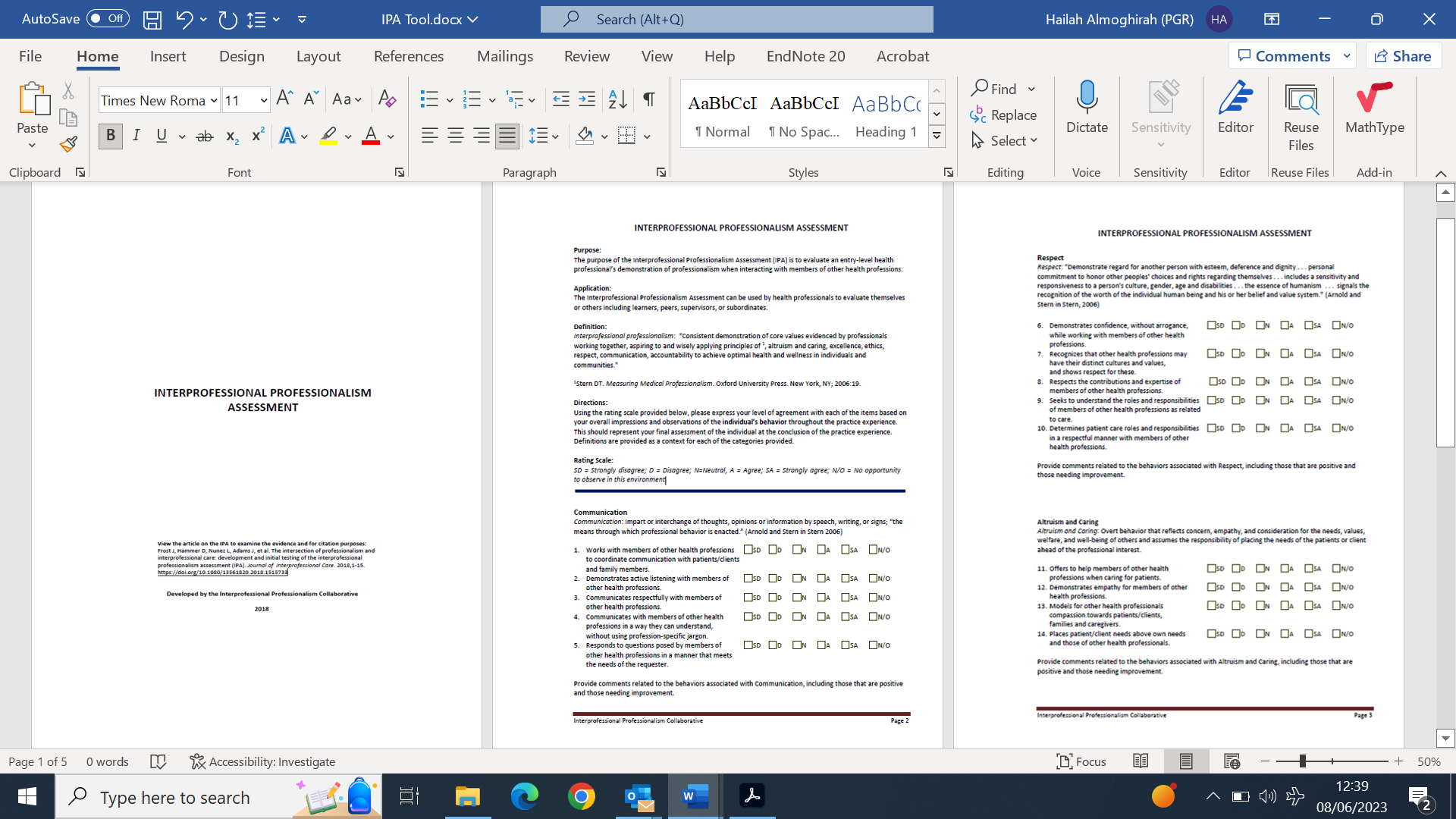


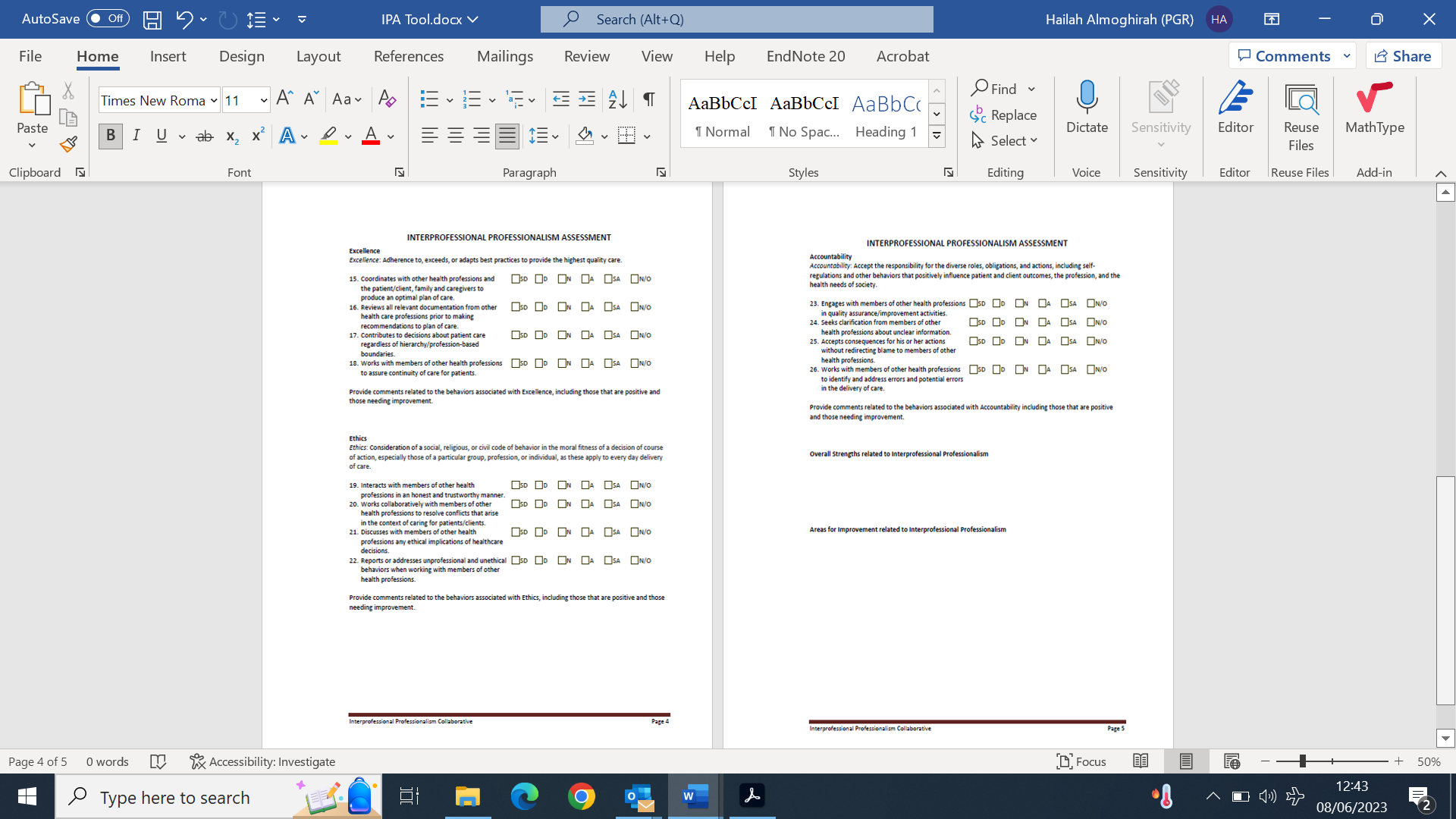


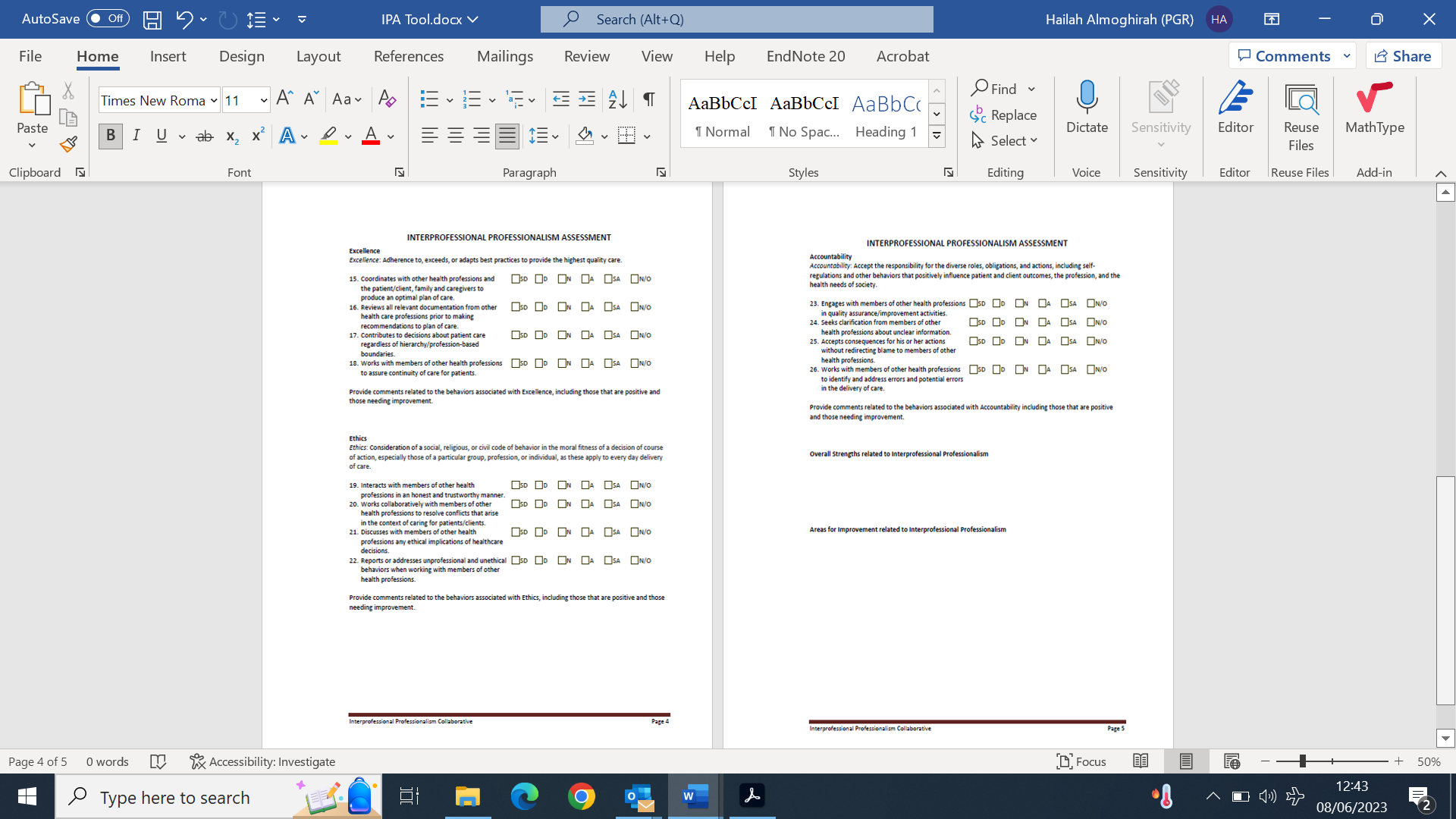
**Appendix S7:** Discharge letter’s rubric used by assessment team

| Quality |  |
| --- | --- |
| The information is descriptive, informative, non-ambiguous and appropriately articulated, e.g. medical terminology is appropriately used, to understand the patient journey and onward care | 10-8 |
| The information is somewhat clear and descriptive with some discrepancies in the patient journey and onward care | 7-5 |
| The information is minimal in description, is ambiguous and does not appropriately describe the patient journey and onward care. | 4-0 |

| Completeness |  |
| --- | --- |
| The information is complete in terms of describing the patient presentation, treatment, medication, directions, referrals and onward care | 10-8 |
| The information is somewhat clear in terms of describing the patient presentation, treatment, medication, directions, referrals and onward care | 7-5 |
| The information is not clear in terms of describing the patient presentation, treatment, medication, directions, referrals and onward care | 4-0 |

| Presentation |  |
| --- | --- |
| The information is provided in a coherent, intelligible and professional manner | 10-8 |
| The information is provided in a somewhat coherent, intelligible and professional manner | 7-5 |
| The information is provided in an incoherent, unintelligible and unprofessional manner | 4-0 |

**Appendix S8:** The Difference on IPA scores between professions

|  | | | | | |
| --- | --- | --- | --- | --- | --- |
| Source | Type III Sum of Squares | Df | Mean Square | F | Sig. |
| Intercept | 971.918 | 1 | 971.918 | 9622.082 | .000 |
| Profession | .033 | 1 | .033 | .322 | .578 |
| Error | 1.616 | 16 | .101 |  |  |

**Appendix S9:** Discharge letter scores improvement over the three IPE sessions and between professions using mixed ANOVA analysis .

| Source | | Type III Sum of Squares | Df | Mean Square | F | Sig. |
| --- | --- | --- | --- | --- | --- | --- |
| DL | Sphericity Assumed | 87.815 | 2 | 43.907 | 6.308 | .005 |
|  | Greenhouse-Geisser | 87.815 | 1.483 | 59.207 | 6.308 | .011 |
|  | Huynh-Feldt | 87.815 | 1.701 | 51.617 | 6.308 | .008 |
|  | Lower-bound | 87.815 | 1.000 | 87.815 | 6.308 | .023 |
| DL * Profession | Sphericity Assumed | 4.111 | 2 | 2.056 | .295 | .746 |
|  | Greenhouse-Geisser | 4.111 | 1.483 | 2.772 | .295 | .681 |
|  | Huynh-Feldt | 4.111 | 1.701 | 2.416 | .295 | .711 |
|  | Lower-bound | 4.111 | 1.000 | 4.111 | .295 | .594 |
| Error(DL) | Sphericity Assumed | 222.741 | 32 | 6.961 |  |  |
|  | Greenhouse-Geisser | 222.741 | 23.731 | 9.386 |  |  |
|  | Huynh-Feldt | 222.741 | 27.220 | 8.183 |  |  |
|  | Lower-bound | 222.741 | 16.000 | 13.921 |  |  |
